# Effects of Two Varieties of *Bacillus thuringiensis* Maize on the Biology of *Plodia interpunctella*

**DOI:** 10.3390/toxins4050373

**Published:** 2012-05-24

**Authors:** Aiko Gryspeirt, Jean-Claude Grégoire

**Affiliations:** 1 Biological Control and Spatial Ecology Laboratory (LUBIES), CP 160/12, Université Libre de Bruxelles, av. FD Roosevelt 50, B-1050 Brussels, Belgium; Email: jcgregoi@ulb.ac.be; 2 Research Funding for Industry and Agriculture (FRIA, Fonds pour la Formation à la Recherche dans l’Industrie et l’Agriculture) 5 rue d’Egmont, B-1000 Brussels, Belgium

**Keywords:** cry toxin, *Bt* maize, *Plodia interpunctella*, biological parameters

## Abstract

On the market since 1996, genetically modified plants expressing an insecticidal toxin (Cry toxin stemmed from *Bacillus thuringiensis*) target several lepidopteran and coleopteran pests. In this study, we assessed the impact of two varieties of *Bt* maize producing different toxins (Cry1Ab or Cry1Fa, respectively) on the biology of a storage pest: *Plodia interpunctella* (Hübner) (Lepidoptera: Pyralidae). The Indianmeal moths were susceptible to both toxins but showed an escape behavior only from Cry1Fa. The weight of females issued from larvae reared on Cry1Ab increased with increasing toxin concentration, but adults of both sexes reared on Cry1Fa had decreased weight. Both toxins increased development time from egg to adult regardless of sex and had no impact on the male adult lifespan. Finally, we recorded a time lag between metamorphosis from the non-*Bt* and the *Bt* diets, which increased proportionally to Cry concentration in the *Bt* diet.

## 1. Introduction

In 1987, the first genetically modified plants expressing their own insecticide, called *Bt* plants, were developed [1,2]. These plants produce at least one Cry protein, a toxin naturally expressed by a gram-positive bacterium: *Bacillus thuringiensis* (*Bt*). After ingestion by an insect, this toxin binds with specific receptors of the midgut epithelium, forming pores in the membrane. The resulting perturbation of the ionic balance leads to swelling and lysis of these midgut epithelial cells and ultimately to death [3,4]. *Bacillus thuringiensis*-based insecticidal products present a highly specific mode of action. The bacterial endotoxin is safe for non-target organisms because the parasporal crystal interacts with a specific receptor in the insect midgut. 

Previous studies have already evaluated the impact of Cry toxins on the biology of insects [5,6,7,8,9,10]. In this work, the effects of lethal and sublethal concentrations of Cry toxins on important components of an insect life cycle were quantified: larval movement, insect avoidance behavior, development time, adult lifespan and weight. In our study we used the Indianmeal moth *Plodia interpunctella* (Hübner), a cosmopolitan and highly destructive storage pest. This insect is an ideal model organism for such studies as it is susceptible to Cry toxins [7,11,12,13,14,15,16], and as *Bt*-resistant strains have already appeared after prolonged toxin application [13,17,18,19]. Furthermore, the species is easy to study in the laboratory as it feeds on stored products (e.g., grains, milled cereal products, and other commodities).

## 2. Materials and Methods

### 2.1. Maize Varieties

We used two distinct *Bt* maize varieties, each directly provided by the producer and containing a gene coding for the production of a different *Bt* protein: respectively MON810 and TC 1507.

YieldGard^®^ grain (Monsanto), produces the Cry1Ab protein derived from *Bacillus thuringiensis* var. *kurstaki* HD-1. The protein expression is regulated by the cauliflower mosaic virus (CaMV) 35S promoter used in the transformation process. The toxin is expressed at an effective dosage for the entire growing season in all plant tissues [11]. The average Cry1Ab protein concentration in MON810 maize is 0.455 μg/g fresh weight tissue (fwt) in the grain [20].

TC 1507 was developed by collaboration between Dow AgroSciences LLC and Pioneer Hi-Bred International, Inc. This event expresses the Cry1Fa protein from *Bacillus thuringiensis* var. *aizawai*, regulated by the maize polyubiquitin promoter. According to Pioneer, from whom we sourced the maize used in this study, Cry1Fa is detectable in the grain with average levels of 90 ng/mg total protein. 

The units for Cry concentrations differ between both varieties (μg/g fwt *vs.* ng/mg total protein in the grain). This lack of standardization hindered direct comparisons between the impacts of these two different events.

A standard non-*Bt* maize grain from biological agriculture (“*Les 4saisons*”, Andrimont-Linea verde Bio maize) was chosen as a control. As *Plodia interpunctella* larvae cannot feed on whole intact kernels, kernels of each maize type were cracked with commercial domestic coffee mills, using separate machines for each of the three maize varieties to avoid contamination. The majority of the particles were 1–2.25 mm in diameter.

### 2.2. *Plodia interpunctella* Strains and Rearing Conditions

The strain of *Plodia interpunctella* used in our experiments was obtained from a laboratory colony maintained at San Joaquin Valley Agricultural Sciences (Parlier). This strain has been reared since the 1960s without any contact with *Bacillus thuringiensis*. 

Mass rearing was made in transparent polystyrene boxes (1.2 litres) with wire netting sealing the top but allowing ventilation. The standard diet (“standard wheat diet”), recommended by Dr. Camilla Ryne of the Laboratory of Chemical Ecology and Ecotoxicology of Lund University (Sweden), was composed by a 150:15:30 g mixture of wheat germ, malted brewers’ yeast and glycerol. All insects were reared at 22 °C, under a 16:8 h (L:D) photoperiod and 70% RH.

### 2.3. Impact of Different Diets on the Biology of *Plodia interpunctella*

Laboratory tests quantified the impact of exposure to lethal and sublethal Cry toxin concentrations on important components of *Plodia interpunctella* life cycle in polystyrene boxes as described above. *Bt* maize and non-*Bt* maize were mixed for 3 min with a domestic mixer to obtain different Cry concentrations in the diet (*Control*: only non-*Bt* maize; *Cry*1*Ab diet*: 0.05–0.09–0.11–0.14–0.16–0.18–0.20–0.23μg Cry1Ab/g fwt in the grain; *Cry*1*Fa diet*: 9–27–45–54–63–72–81–90 Cry1Fa ng/mg total protein in the grain). For each diet, 100 g of cracked maize was mixed with 20 g of glycerol. 50 newly laid eggs (<24 h old) were placed in each box. The eggs were obtained by placing newly emerged adults of *Plodia interpunctella* into a Petri dish lined with filter paper. Eggs laid overnight were collected with an entomological needle and placed in a rearing treatment box. There were 3 replicates per concentration and per maize variety.

Three times a week, the numbers of surviving adults emerging from each treatment and the numbers of days required from the egg to adult emergence were counted, the adults were weighed and sex was determined using morphological features [21]. At emergence, each adult was isolated in an individual empty cup to determine adult life duration. In this bioassay, mortality induced by the toxin was based upon comparison of adult emergence between the different diets.

### 2.4. Choice Experiments: Larval Preference between Two Diets

Larval preference between the two diets was tested. Each experimental unit comprised a clear polystyrene box (9 cm long × 5 cm wide × 4.5 cm high) separated into two parts by a 0.5 cm high piece of cardboard.

The mobility of the larvae in this experimental system was first measured by following their diffusion on a neutral substrate. Each side contained non-*Bt* maize and twenty larvae were placed in one side (3 replicates). Twenty-four h later, the distribution of the larvae was recorded.

Larval preference between the different maize varieties was tested. In the choice-test, MON810 maize or TC 1507 maize was put in one area and non-*Bt* maize in the other area. As a control, non-*Bt* maize was put in the two areas. Fifteen 3rd or 4th instar larvae were placed in each side and their distribution was recorded 24 h later. We used older larvae because their transfer to the experimental system was easier and their mortality was lower. There were five replicates per experiment.

### 2.5. Data Analysis

To characterize the susceptibility of *Plodia interpunctella* to each toxin, the regression equations between larval mortality and toxin concentration were calculated as well as the determination coefficients R^2^. These data were adjusted for mortality with Abbott’s correction. Since our control mortalities were high, it became difficult to distinguish between the effects of the diet and that of the added toxin. Chi^2^ tests were used to check if susceptibility to the toxins was linked to sex.

In the experiments designed to examine larval preference between two diets, G-tests comparing the distribution of the larvae between these two areas were performed. 

Before any statistical analysis comparing means (development duration, weight, adult stage duration), normality was tested by a Kurtosis test and homoscedasticity by a Levene test. When these two conditions were met, a Student’s t test was performed for comparing two means, and an ANOVA was used for comparing more than two means. When the conditions were not met, non-parametric tests (Mann-Whitney or Kruskal-Wallis), were applied and, if necessary, pairwise comparisons were performed with the Bonferroni correction. The correlation between two parameters was assessed by calculating Pearson’s correlation (or Kendall’s correlation if normality was not met). Finally the link between Cry concentration in the diet and several biological parameters was also calculated by regression analysis and calculation of the determination coefficient R^2^. All analyses were made using R [22].

## 3. Results and Discussion

Our laboratory-based experimental system had several benefits over a field-based system, in that it was temporally and environmentally stable (including control over temperature, luminosity, toxin concentration, and toxin exposure). Furthermore, the short (~27 days) and continuous life cycle of *Plodia interpunctella*,combined with high fecundity in our experimental organisms, rapidly provided several successive generations, allowing for efficient testing of our hypotheses. 

### 3.1. Susceptibility of *Plodia interpunctella* to the Cry Toxins

**Figure 1:**
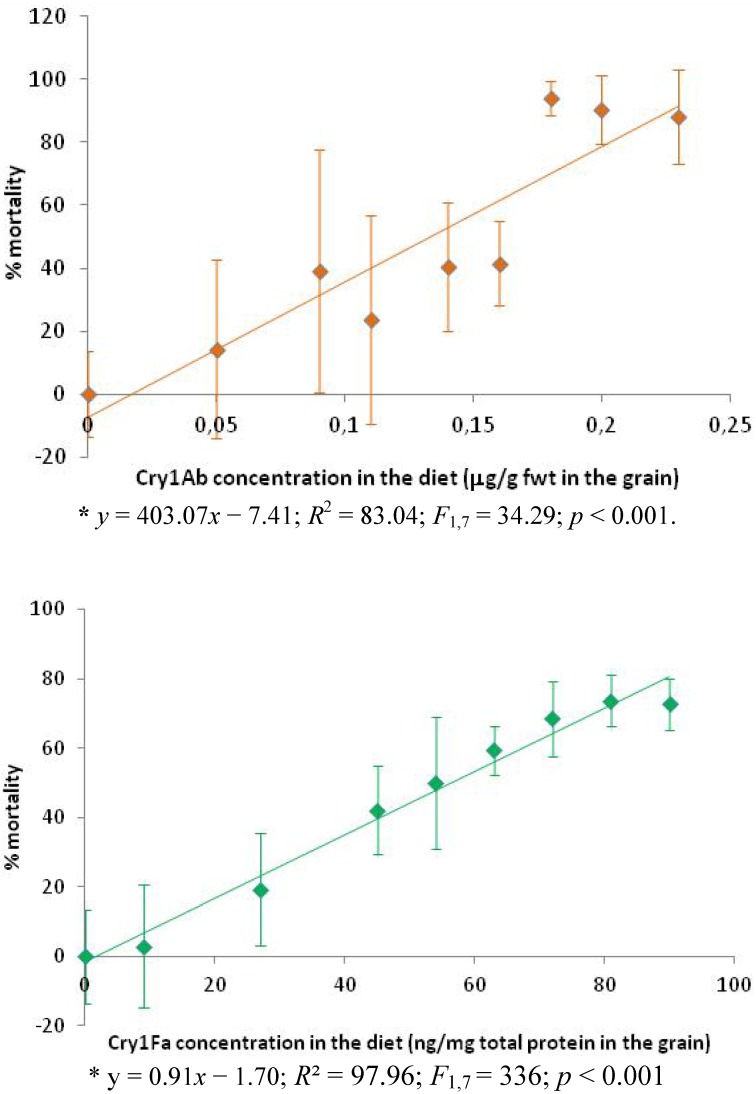
*Plodia interpunctella* susceptibility to Cry concentration in the rearing diet (% survival insects).

Susceptibility to the toxins was independent of sex (Table 1). The sex ratio was balanced for the control (*χ*^2^_1 _= 1.68; *p* = 0.194) and for the samples reared on the two different toxins (*χ*^2^_1Cry1Ab_ = 0.32; *p* = 0.569 and *χ*^2^_1Cry1Fa_ = 2.95; *p* = 0.086). The susceptibility of the larvae to both Cry toxins was demonstrated: mortality increased with the presence of the toxins in the diet (see Figure 1). We described this with a significant linear regression equation (Cry1Ab: *R*² = 83.04 (*F*_1,7 _= 34.29; *p* < 0.001) ; Cry1Fa: *R*² = 97.96 (*F*_1,7 _= 336; *p* < 0.001)). 

We observed 72.62% mortality with a diet composed by 100% Cry1Fa maize (90 ng Cry1Fa/mg total protein in the grain). In contrast, 88.10% of the susceptible insects were killed on a diet composed of only 50% Cry1Ab maize (0.23 μg Cry1Ab/g fwt in the grain). This suggested that Cry1Ab maize can more effectively control the Indianmeal moth in stored grain. This observation is consistent with the findings of Oppert *et al.* [10] that *Plodia interpunctella* is more sensitive to the Cry1Ab toxin than to the Cry1Fa protoxin.

Our results were also consistent with other studies evaluating the impact of Cry toxins on *Plodia interpunctella* [7,10,14], on *Helicoverpa zea* [23,24] and on *Pectinophora gossypiella* [25]. The recorded variability of susceptibility on the different diets can be explained by the possible heterogeneity of the maize particles (*Bt* and non-*Bt*) both in size and in composition. Indeed, our diet was not composed of the finest cracked maize (<1 mm) but by larger particles of cracked maize (1–2.25 mm), as our preliminary experiments suggested that *Plodia interpunctella* larvae cannot develop in powder diet. After grinding, some particles would be principally endosperm or principally embryo particles and other particles would be portions of both endosperm and embryo. These heterogeneities would also likely cause heterogeneity in the level of Cry protein exposition. This artifact was, however, not controllable.

Insect survival was low in our control diet (56.0% ± 7.6%). Such low survivorship rates have been recorded in previous studies involving *P. interpunctella*: 67.5% ± 7.5% [7] or 35.4% [12]. The high mortality may have been the result of one or several possible factors, such as egg transfer techniques, diet quality or experimental stress. For example, whereas in previous work by Liu *et al*. [25], eggs of *Plutella xylostella* were collected on pieces of paper towel and inserted under a bract of individual cotton bolls (giving a non-*Bt* larval survival of 24.0%), our own technique of egg transfer from the egg-laying arenas to the experimental diets using an entomological needle could potentially have damaged more eggs. The high mortality recorded on our non-*Bt* diet could also be induced by the maize diet, as maize is not an optimal food for *Plodia interpunctella* because it produces a natural pesticide (DIMBOA) as secondary metabolite [26]. 

**Table 1:** Adult sex ratio of *Plodia interpunctella* in relation to the rearing diet.

Rearing diet	*n*	♀:♂	*χ*²	df	*p*
**Non-*Bt* maize diet**	152	0.45:0.55	1.68	1	0.194
**Standard wheat diet**	521	0.48:0.52	0.85	1	0.358
**Cry1Ab diet**	308	0.52:0.48	0.32	1	0.569
**Cry1Fa diet**	369	0.54:0.46	2.95	1	0.086

### 3.2. Choice Experiments: Larval Preference between Two Diets

In our larval diffusion experiments the distribution was uniform between both non-*Bt* diets after twenty-four h (*G*_1_ = 1.67; *p* = 0.196), indicating that the larvae moved without constraints in the experimental system (see Table 2). Larval distribution was also uniform in the control experiment composed of non-*Bt* maize (*G*_1_ = 0.48; *p* = 0.486) and in the Cry1Ab toxin experiment (*G*_1_ = 0.45; *p* = 0.501). Thus, in our experimental system, larvae did not avoid the Cry1Ab toxin contained in the diet. However, larvae significantly escaped from the Cry1Fa zone to take refuge in the non-*Bt* zone (70% of the larvae were in the non-*Bt* maize zone; *G*_1_ = 2.70; *p* < 0.001). In this case, larval behavior was influenced by the presence of the Cry1Fa toxin in the diet, suggesting that, according to the strain of the Cry toxin, the larvae reacted differently.

A similar avoidance behavior has previously been recorded for *Heliothis virescens* (Fabricius) larvae (Lepidoptera: Noctuidae). Resistant and susceptible strains avoided moderate and high concentrations of the toxins (Dipel2X or purified HD-73 σ-endotoxin). At the lowest Dipel2X concentration, only the susceptible strain avoided the diet containing Dipel [27]. In another experiment, the movements of *Heliothis virescens* were wider on *Bt* cotton plants and the interplant movement was increased in relation to the age of the larvae [28]. Similarly, neonates of *Ostrinia nubilalis* have been found to be able to detect *Bt* in corn tissue within an hour of hatching, and were measured to escape 1.78 times more frequently from a *Bt* maize plant than from a non-*Bt* plant [29].

This escape behavior can be explained by the mode of action of the Cry toxins. Unlike many synthetic insecticides going through the integument and acting on the insect nervous system, Cry toxins have to be ingested [3]. Damage (swollen microvilli, loss of the outer cell wall membrane of the gut epithelial cells, disintegration into individual organelles, *etc*.) has been observed on gut epithelial 11 min after toxin ingestion [30], triggering two key behavioral changes. First, the larvae stop feeding and anorexia may be induced within about 24 h. If the dose of Cry toxin ingested is not lethal, midgut restoration may subsequently occur [30]. Following recovery from acute toxic effects, larvae increase their tendency to travel, taking them away from the just ingested Cry diet and creating new encounters with food. This mechanism thus involves neither “avoidance” of toxic foods nor active search for food that does not contain toxins. Larvae have not been noted to voluntarily discriminate between *Bt* and non-*Bt* foods before ingestion [29,31,32]. 

Cry1Ab toxin was also toxic for *Plodia interpunctella*, as proven previously, but an escape reaction of the larvae from the Cry1Ab area to the refuge was not noted, probably because the individuals were counted too early (24 h after the experiment started). At the time of counting, the insects were at the end of their anorexia phase and had probably not yet begun their walking phase. 

**Table 2:** Larval distribution (%) between different zones.

			Homogeneity of the replicates			Comparison of the larval distribution		
		(%)	*G*-Test	df	*p*	*G*-Test	df	*p*
**Larval diffusion**	point of departure	58.0	1.87	2	0.403	1.67	1	0.196
	adjacent zone	42.0						
**Control**	non *Bt* zone	47.0	3.02	4	0.406	0.48	1	0.486
	Non *Bt* zone	53.0						
**MON810 maize case**	Cry1Ab zone	52.8	4.86	4	0.301	0.45	1	0.501
	non *Bt* zone	47.2						
**TC 1507 maize case**	Cry1Fa zone	30.4	2.78	4	0.301	2.70	1	<0.000
	non *Bt* zone	69.6						

### 3.3. Impact of the Diet on the Adult Weight

Weight is an important biological parameter because it can be considered as an indirect, but easily measurable, indicator of insect fitness regarding Cry toxins and is correlated with other biological parameters (e.g., fecundity) [33]. 

Our data regarding both sexes were not pooled because females were heavier than males in the control (Mann-Whitney test: U_68;84_ = 964.50; *p*< 0.001) (see Table 3). 

There was a significant and positive linear regression between the Cry1Ab concentration in the diet and female adult weight (*R*^2^ = 69.01; *F*_1,5_ = 10.61; *p* < 0.001). Moreover, females reared on diets containing 0.05, 0.14, 0.16 or 0.18 μg Cry1Ab/g fwt in the grain were significantly heavier (up to 1.3×) than those in the control (*p* < 0.05 for the pairwise comparison with the Bonferroni correction). This female weight increase was not correlated with any increase in development time. In the control diet there was no positive correlation between these two parameters (Kendall’s correlation tau = −0.03; *p* = 0.712). There was also no significant correlation for the insects reared on the Cry1Ab diet (females, Pearson’s correlation: *r* = 0.056; *p* = 0.955). The impact of the Cry1Ab toxin on male weight was not so pronounced: no significant linear regression (*R*^2^ = 4.09; *F*_1.5_ = 0.21; *p* = 0.667) but the males reared on 0.11 μg Cry1Ab/g fwt in the grain were heavier than the males from the control (*p* = 0.036 for pairwise comparison with Bonferroni correction).

Although there was no significant linear regression between the adult weight and the Cry1Fa toxin concentration (females, *R*^2^ = 23.88; *p* = 0.181; males, *R*^2^ = 18.76; *p* = 0.244), we recorded a significant female adult weight decrease at intermediate concentrations (loss of 50% of the weight on 54 ng Cry1Fa/mg total protein in the grain; *p* < 0.001 with the Bonferroni correction for pairwise comparison with the control). Similar observations were made for the males: with intermediate concentrations the weight decrease was maximal (weight loss of 50% compared to control and *p* < 0.001 with the Bonferroni correction for pairwise comparison between 54 ng Cry1Fa/mg total protein in the grain and the control). This weight decrease could be explained by food avoidance by the larvae reared on Cry1Fa diets. Indeed, our diet choice experiments showed significant escapes from the diets containing Cry1Fa. 

Cry toxins therefore had inconsistent impacts on the adult weight of *Plodia interpunctella*. Adult weight can be affected by a decrease of food intake. Susceptible larvae fed less on a treated diet than on an untreated diet [34]. They presented symptoms of anorexia when they ingested a Cry toxin [30] as proven by the decrease of dry frass on a *Bt* diet [27]. 

Previous work has, however, suggested that resistant larvae might use Cry toxins as a supplementary food protein [35], although this idea was questioned by Tabashnik & Carriere [36] because the reduction of susceptibility may not be sufficient to completely overcome the negative effects of the toxin.

**Table 3:** Adult weight (mg) and adult lifespan (days) of *Plodia interpunctella* in relation to the sex and the rearing diet. Standard errors are provided in parentheses.

Concentration in the rearing diet		Adult weight (mg)				Adult lifespan (day)			
		N	Female	N	Male	N	Female	N	Male
Cry1Ab g/g fwt in the grain	0	68	12.15 (3.35)	84	8.45 (1.45)	65	20.00 (4.82)	83	20.00 (6.96)
	0.05	41	14.90 (2.20)	31	9.02 (1.15)	41	20.97 (3.92)	31	21.07 (5.61)
	0.09	28	14.17 (3.21)	22	8.96 (0.85)	28	20.91 (4.82)	22	20.91 (5.90)
	0.11	30	13.63 (3.21)	31	9.38 (1.81)	30	20.48 (4.27)	31	20.58 (3.68)
	0.14	25	15.00 (3.15)	19	9.26 (1.74)	25	20.41 (4.60)	17	20.41 (3.84)
	0.16	15	15.24 (2.95)	28	9.98 (2.52)	14	17.85 (5.36)	26	17.85 (5.86)
	0.18	11	16.08 (2.64)	9	7.44 (1.66)	11	17.00 (2.71)	9	17.00 (4.58)
Cry1Fa ng/mg tot prot in the grain	0	68	12.15 (3.35)	84	8.65 (1.55)	65	20.20 (4.82)	83	20.20 (6.96)
	9	44	9.17 (2.23)	41	5.96 (1.38)	44	21.22 (6.47)	41	21.22 (6.27)
	27	46	7.60 (2.13)	28	4.39 (1.29)	46	18.41 (5.73)	27	18.41 (6.86)
	45	34	7.94 (2.63)	19	4.80 (1.37)	34	20.26 (5.68)	19	20.36 (6.43)
	54	25	6.65 (1.75)	22	4.43 (1.45)	25	17.77 (5.38)	22	17.87 (6.25)
	63	12	7.69 (1.33)	18	4.85 (1.23)	12	19.17 (5.59)	18	19.27 (6.58)
	72	15	8.63 (1.92)	15	5.86 (1.03)	15	22.93 (5.97)	15	22.93 (4.50)
	81	8	7.76 (2.40)	10	5.10 (1.12)	8	21.70 (5.71)	10	21.70 (5.52)
	90	10	9.22 (3.18)	10	5.92 (0.90)	10	23.80 (7.54)	10	23.80 (4.05)

### 3.4. Impact of Bt Maize on the Life Cycle of *Plodia interpunctella* (from Egg to Adult Emergence-Adult Lifespan)

**Figure 2:**
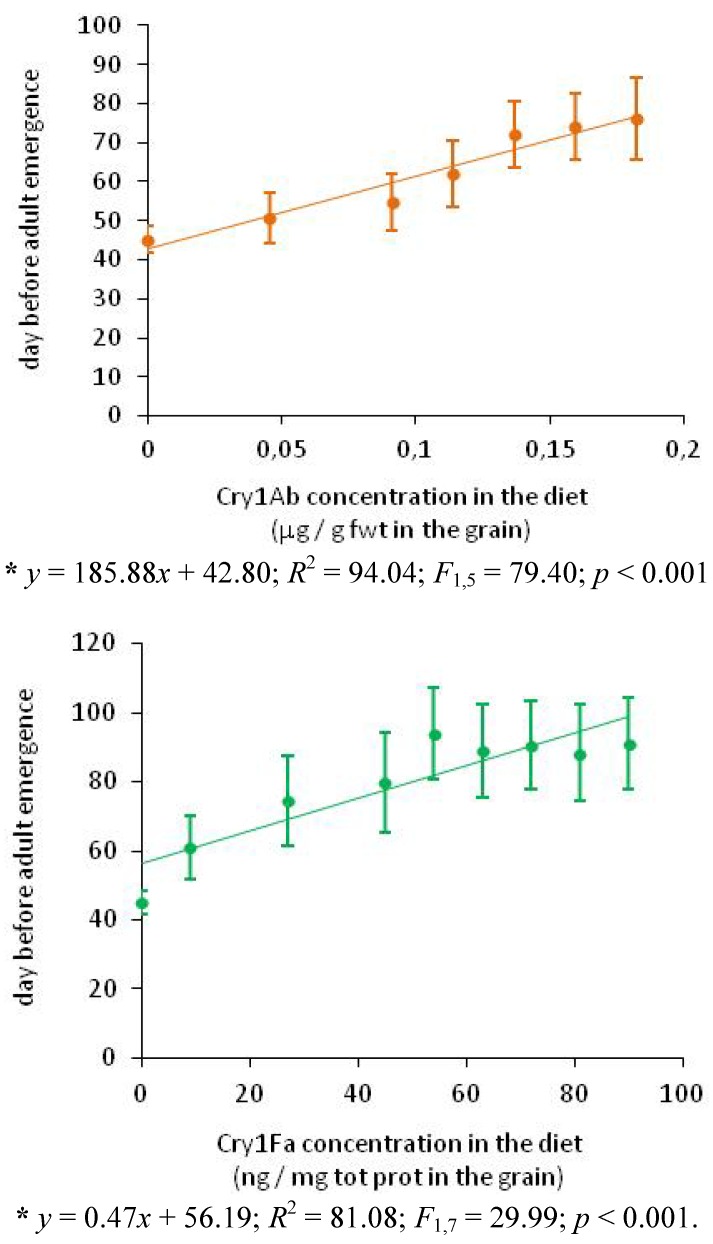
Number of days (mean ± SD) between egg stage and adult emergence for *Plodia interpunctella* in relation to the Cry concentration in the rearing diet.

The Cry toxins included in the diet had a significant impact on development duration (from the egg to adult emergence*)*. Females and males were pooled as both sexes presented the same development duration in the control (Mann-Whitney test: U_68, 84_= 2413.50; *p* = 0.097). Development time grew significantly and linearly in relation to the Cry1Ab concentration in the diet (*R*^2^ = 94.04; *F*_1,5_ = 79.40; *p* < 0.001). With 0.18 µg Cry1Ab/g fwt in the grain there was a 1.7 increase in developmental time. Similar conclusions can be made for the Cry1Fa toxin (*R*^2^ = 81.08; *F*_1,7 _=29.99; *p* < 0.001). (see Figure 2). Developmental time was doubled with high Cry1Fa concentrations (> 54 ng Cry1Fa/mg total protein in the grain).

As adult lifespan was significantly longer among the males in the control, the data for both sexes were not pooled for all analyses (Mann-Whitney test: U_65, 83_ = 1082.50; *p* < 0.001) (see Table 3). 

The adult lifespan of the females reared on Cry1Ab linearly increased in relation to the toxin concentration (*R*^2^ = 76.27; *F*_1,5_ = 16.07; *p* = 0.010). On the other hand, Cry1Ab and Cry1Fa had no significant impact on the male adult lifespan (Cry1Ab: *R*^2^ = 43.38; *F*_1,5_ = 3.83; *p* = 0.107; Cry1Fa: *R*^2^ = 20.95; *F*_1,7 _= 1.85; *p* = 0.215). Finally, although there was no significant linear regression between the adult lifespan of the females and the Cry1Fa toxin concentration (*R*^2^ = 36.70; *F*_1,7 _= 4.07; *p* = 0.367), we recorded a significantly longer lifespan for females reared on 50 ng Cry1Fa/mg total protein in the grain than in the control (Kruskal-Wallis test: H_8 _= 23.70; *p* = 0.003 and pairwise comparison control: *p* = 0.002). 

Similar increases in life cycle duration on *Bt* maize grain have already been demonstrated on *Plodia interpunctella* [7,12], *Pectinophora gossypiella* [23] and *Helicoverpa zea* [23,24,37]. This principally concerned the larval stages [24], probably because the *Bt* toxin suppressed feeding [30], see also our own results above, as a consequence of which larvae needed more time to develop. The lengthening of larval development delayed pupation and induced higher mortality by longer exposure to natural enemies or abiotic mortality factors [17]. Moreover, overwintering mortality could also be increased because insects might not reach the right stage for diapausing at the end of the growing season [25].

### 3.5. Impact of the Bt Maize on the Percentage of Adults Available for Random Mating

**Figure 3:**
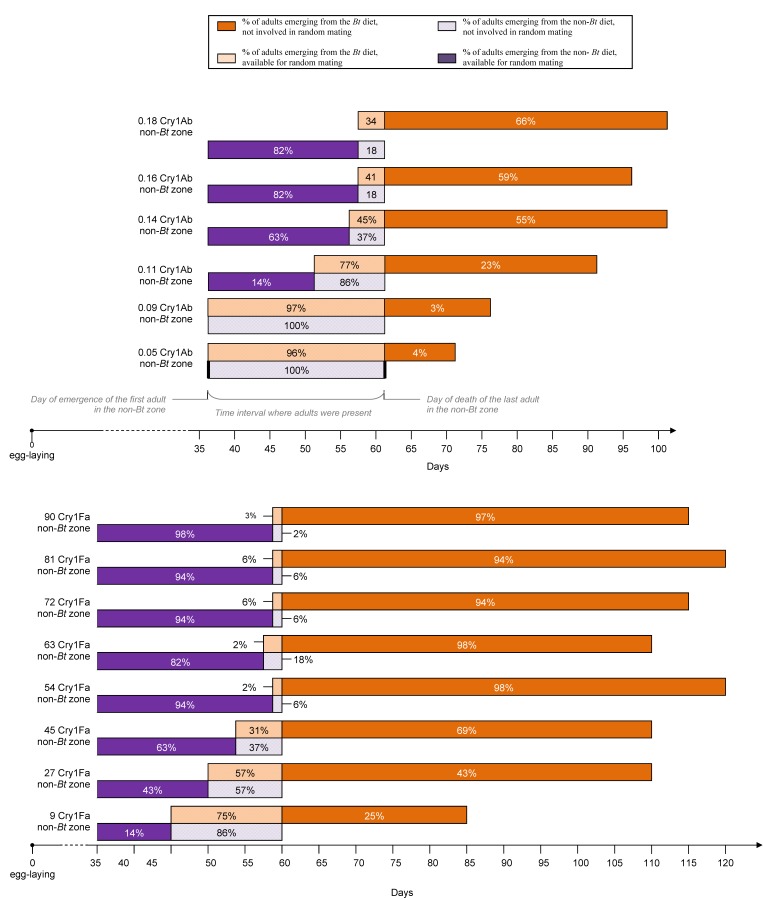
Adults available for random mating. We considered the day of emergence of the first adult and the day of death of the last adult in a same zone (e.g., the 0.18 Cry1Ab zone). We calculated a similar time interval for the non-*Bt* zone. The percentage of adults available or not available for random mating was calculated using the overlapping time interval between the *Bt* and non-*Bt* zones and the adult density.

**Figure 4:**
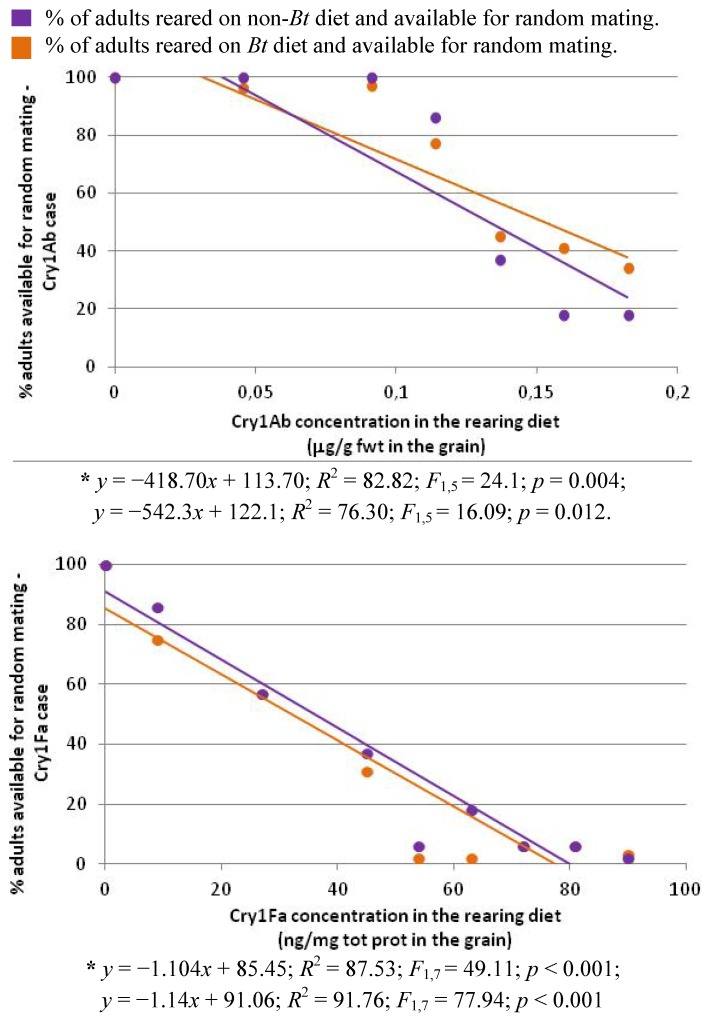
Percentage of adults from the *Bt* zone and the non-*Bt* zone available for random mating in relation to the Cry toxin concentration in the rearing diet

The percentage of adults emerging simultaneously from the non-*Bt* diet and from the *Bt* diet was considered as the proportion of insects available for random mating. To calculate this indicator, we took into account the interval between the day of the emergence of the first adult in a zone and the day of the death of the last adult in this zone. The same time interval was calculated for the non-*Bt* diet. The percentage of adults available for random mating corresponded to the proportion of adults from the non-*Bt* and from the *Bt* diet present at the same time (see Figure 3). For both toxins, we observed a significant and negative linear regression between toxin concentration and the percentage of adult available for the random mating (see Figure 4). The Cry toxin induced a temporal separation between adults emerging from the *Bt* diet and the non-*Bt* diet. With 90 ng/mg total protein in the diet, only 2% of the “*Bt* zone” was available for random mating, with 2% of adults from the “non-*Bt* zone”.

This time lag of adult emergence between the Bt zone and the refuge zone increased with the amount of toxin in the diet, resulting in a growing asynchrony between adult emergence in the two zones. Similar observations have been made by other authors [37,38,39,40]. This asynchrony increases the probability of resistant adults born in the *Bt* zone mating together, weakening one of the main assumptions of the HD/R strategy (random mating independently of the zone and of the genotype) and resulting in faster development of resistance [9]. If this laboratory model is applicable to field conditions, one needs to consider the impact of delayed insect development on the long-term success of the HD/R strategy. When the impact of the Cry diet on the availability of adults for mating was evaluated, it was supposed that fecundity was constant in time. However, delayed mating in *Plodia interpunctella* influences the number of spermatophores transferred, the number of eggs laid, egg viability, as well as the ability of males to inseminate females [41]. It would be interesting to study the variation in fecundity in relation to diet and age, in order to distinguish the contribution of each adult in the mating. 

The presence of Cry toxin in the rearing diet may not have been the only factor that influenced diet choice, adult weight and development duration. We used three different varieties of maize: one *Bt* maize seed, one *Bt* maize grain and one non-*Bt* maize grain. It is possible that there was a difference from one maize variety to the next in kernel hardness or composition. Differences in kernel types, composition or texture would have an impact on their grinding characteristics and thus on particle size in the diet and on the amount of consumption by the larvae. Similar remarks can be made about differences in life cycle duration between maize control and a standard wheat diet. Wheat is considerably higher in protein and lower in lipid and starch than maize (see http://www.fao.org/docrep/T0395F/T0395F02.htm and information concerning the nutritional composition of the germ wheat provided by the firm). These differences could be explained by the composition of the diet but also by its humidity and moisture [42].

These experiments were designed to increase our knowledge of the impact of Cry toxins on the biology of insect pests. We hope that our study will contribute to the control of larvae of *Plodia interpunctella*, a worldwide insect pest of stored products and perhaps the most economically important pest of processed food commodities in stored *Bt* grains [43].
